# Supplementary material for: Sulfonated tryptanthrin anolyte increases performance in pH neutral aqueous redox flow batteries
Source: Commun Chem. 2021 Jun 11;4:89. doi: 10.1038/s42004-021-00523-0 (PMC9814137; doi:10.1038/s42004-021-00523-0)
Supplement: Supplementary file 2 — Supplementary Information [file 42004_2021_523_MOESM2_ESM.pdf]

Supplementary Information

Sulfonated tryptanthrin anolyte increases performance in pH neutral aqueous redox flow batteries

Daniela Pinheiro[1], Marta Pineiro[1] and J. Sérgio Seixas de Melo[1*]

[1] University of Coimbra, CQC, Department of Chemistry, Rua Larga, 3004-535 Coimbra, Portugal

*e-mail: sseixas@ci.uc.pt

Keywords: Redox flow batteries, Neutral pH aqueous redox flow batteries, Tryptanthrin sulfonic acid, all-organic

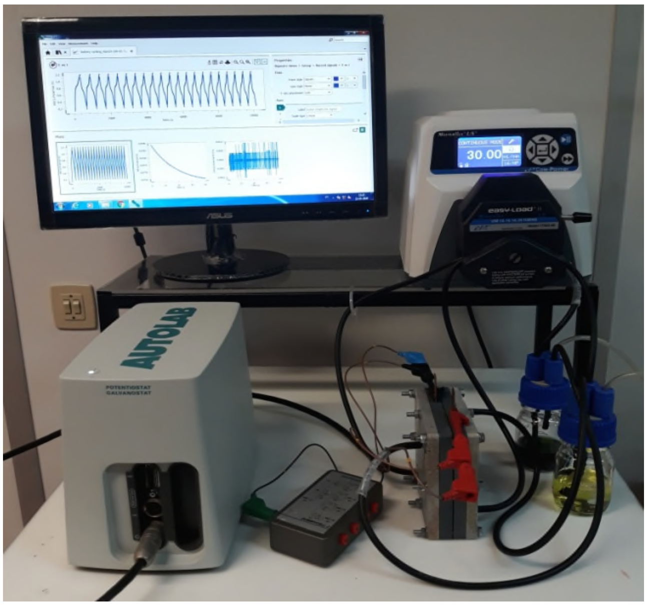

Fig. SI1 Picture of the set-up used for the study of neutral pH AORFB cells.

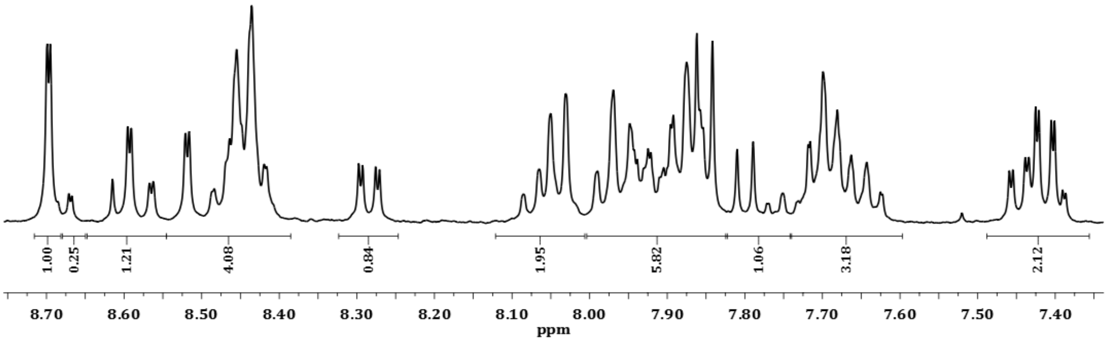

Fig. SI2 ^{1}H NMR ($CDCl_3$) of the reaction crude obtained from chlorosulfonation of TRYP.

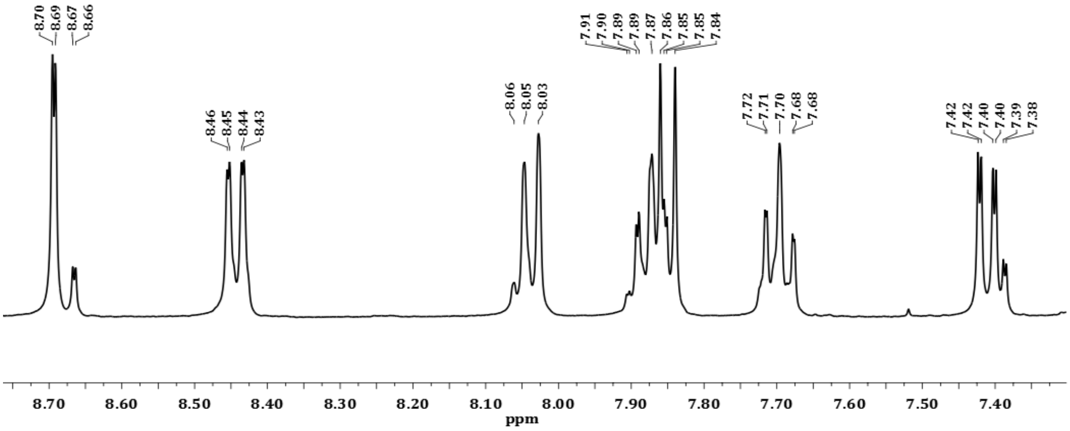

Fig. SI3 ^{1}H NMR of TRYP-8SO_3H and TRYP-2SO_3H in $CDCl_3$.

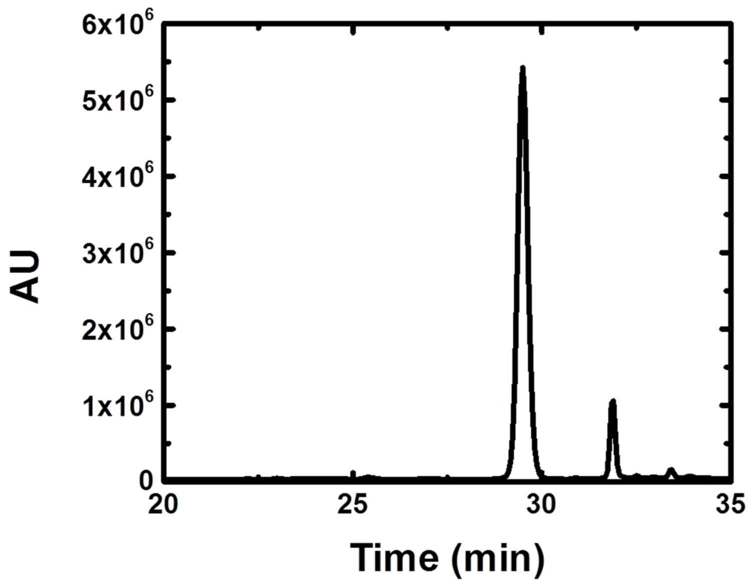

Fig. SI4 Chromatogram of the reaction crude resulting from tryptanthrin sulfonic acid synthesis.

Table SI1. Retention time, area and percentage of total chromatographic area.

DAD 381- 400 nm	Results			
**Retention Time**	**Area**	**Area %**	**Height**	**Height %**
**3.680**	234832	0.15	27774	0.37
**4.267**	1129572	0.73	116514	1.55
**5.173**	1637207	1.06	121922	1.62
**6.827**	72864	0.05	3863	0.05
**8.747**	7518711	4.87	145427	1.94
**10.773**	4975779	3.22	81805	1.09
**12.000**	5274805	3.42	82938	1.10
**13.120**	2218462	1.44	46606	0.62
**14.293**	1305178	0.85	24279	0.32
**15.520**	755561	0.49	15662	0.21
**17.387**	1671515	1.08	16532	0.22
**18.347**	644894	0.42	14526	0.19
**19.200**	1763264	1.14	40060	0.53
**20.907**	244849	0.16	6460	0.09
**22.240**	669427	0.43	15465	0.21
**22.987**	490338	0.32	22825	0.30
**23.520**	364970	0.24	14880	0.20
**23.947**	401685	0.26	13607	0.18
**24.480**	526227	0.34	13783	0.18
**25.387**	918265	0.60	32298	0.43
**26.827**	427097	0.28	9689	0.13
**29.493**	106616994	69.1	5392059	71.83
**30.880**	206173	0.13	16261	0.22
**31.893**	11300561	7.32	1020210	13.59
**32.533**	504208	0.33	39953	0.53
**32.960**	400501	0.26	22198	0.30
**33.440**	1394467	0.90	118009	1.57
**33.920**	633397	0.41	31363	0.42
**Totals**	154301803	100	7506968	100

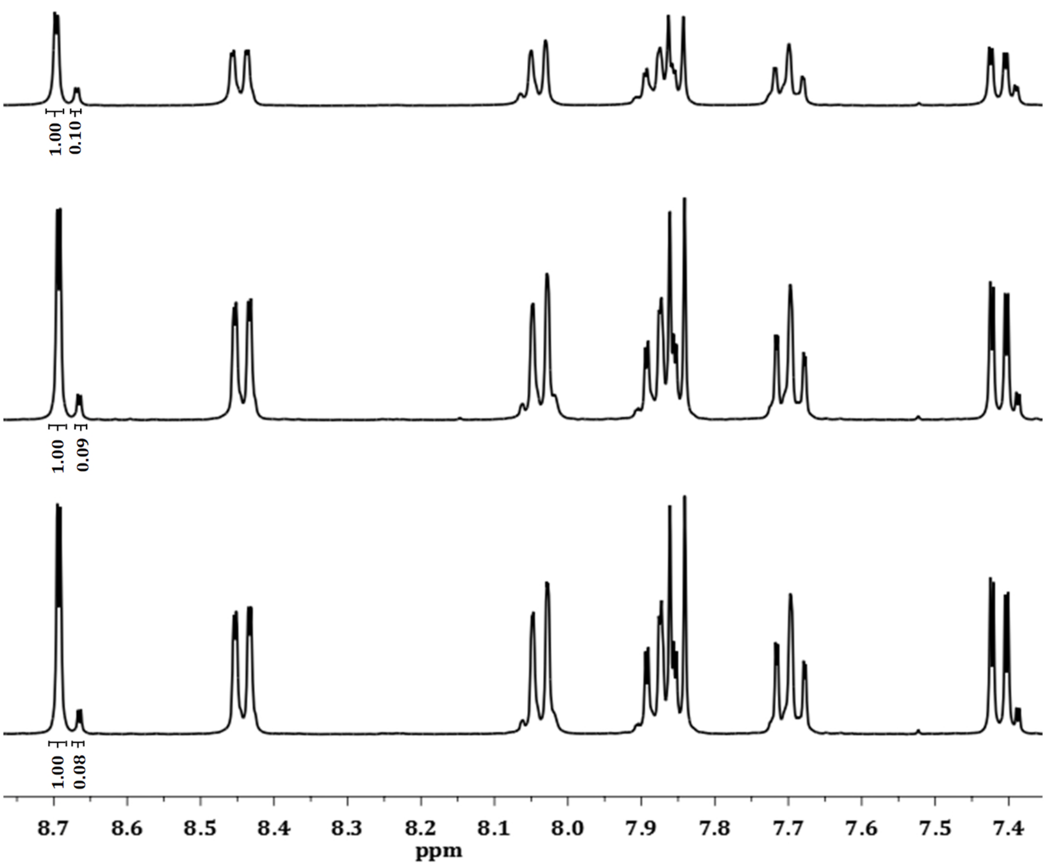

Fig. SI5 ^{1}H NMRs of TRYP-8SO_3H and TRYP-2SO_3H in $CDCl_3$ of three reactions under the same conditions.

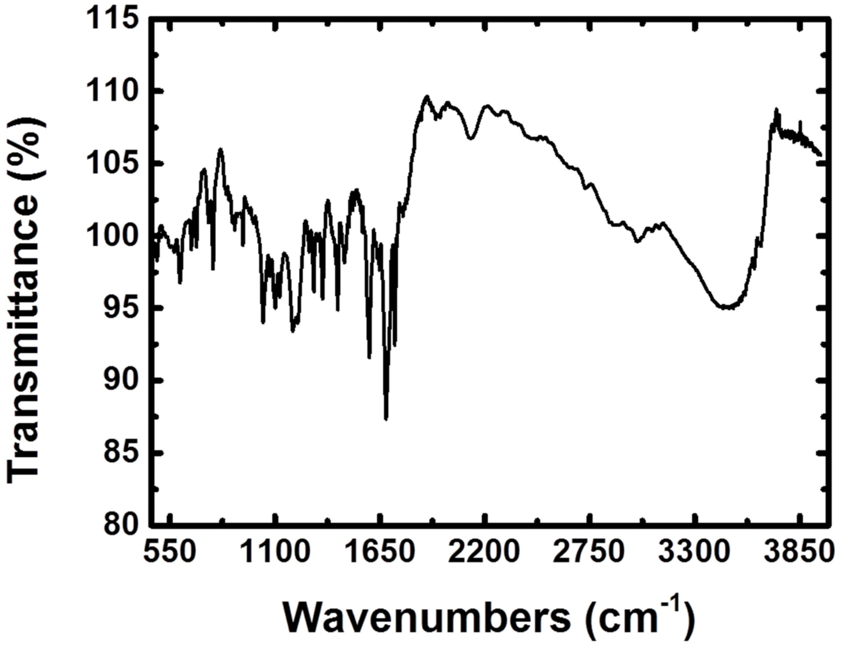

Fig. SI6 IR spectra of tryptanthrin sulfonic acid.

Mass Spectrum Molecular Formula Report

Analysis Info

Analysis Name	D:\Data\UCoimbra\TPMTPM33095104000004.d
Method	Tune_Neg_50_1350.m
Sample Name	**TRYPSO3H**
Comment	ESI -
Acquisition Date	7/22/2020 9:41:48 AM
Instrument	micrOTOF

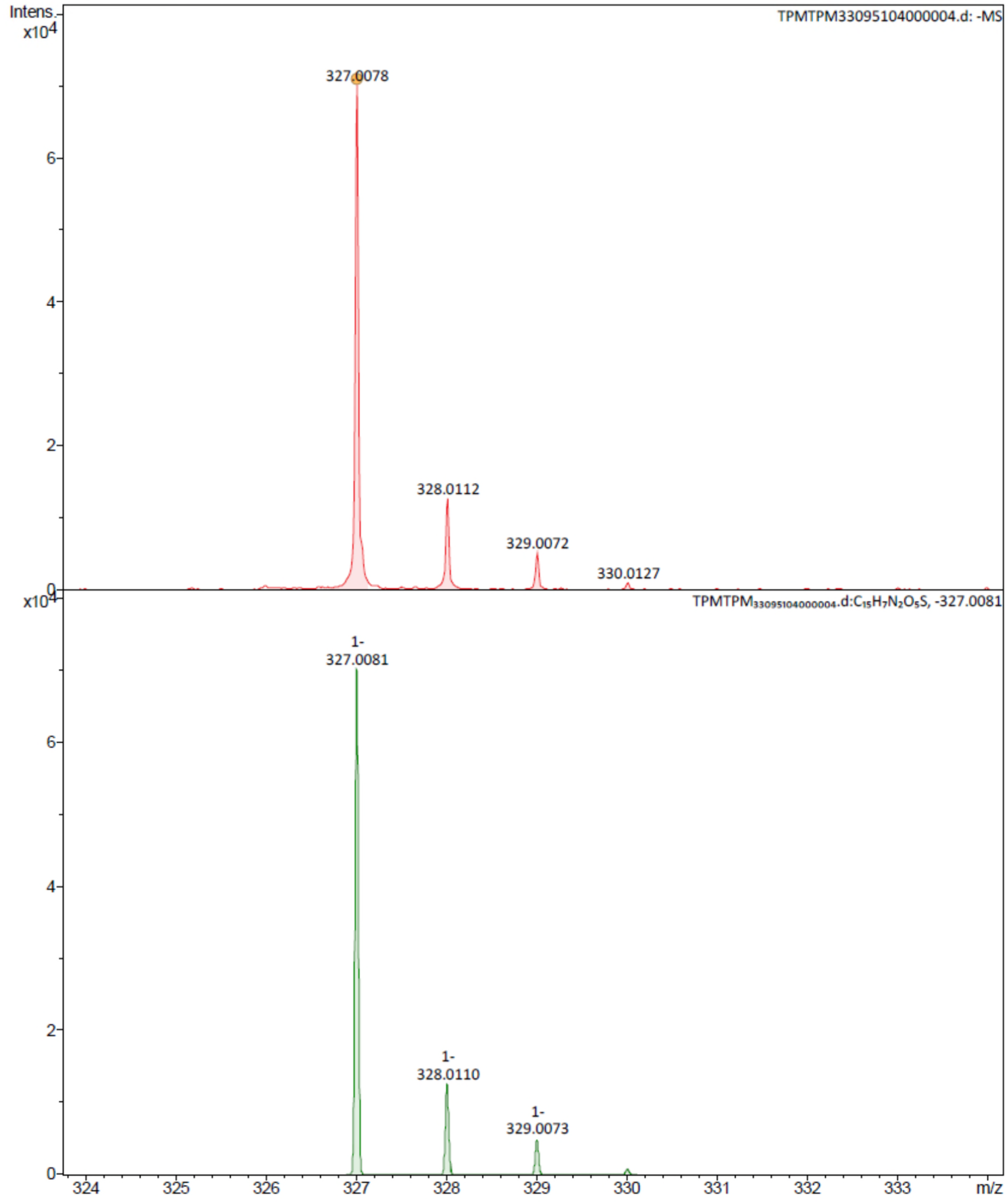

Fig. SI7 High resolution mass spectrum of tryptanthrin sulfonic acid.

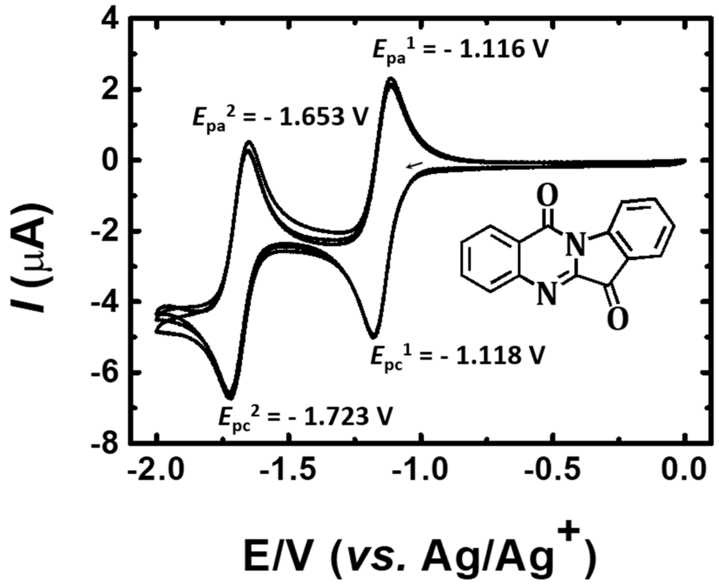

Fig. SI8 Cyclic voltammogram of 1.0 mM of TRYP in MeCN solution, with saturated N_2, υ = 50 mV s^{-1}.

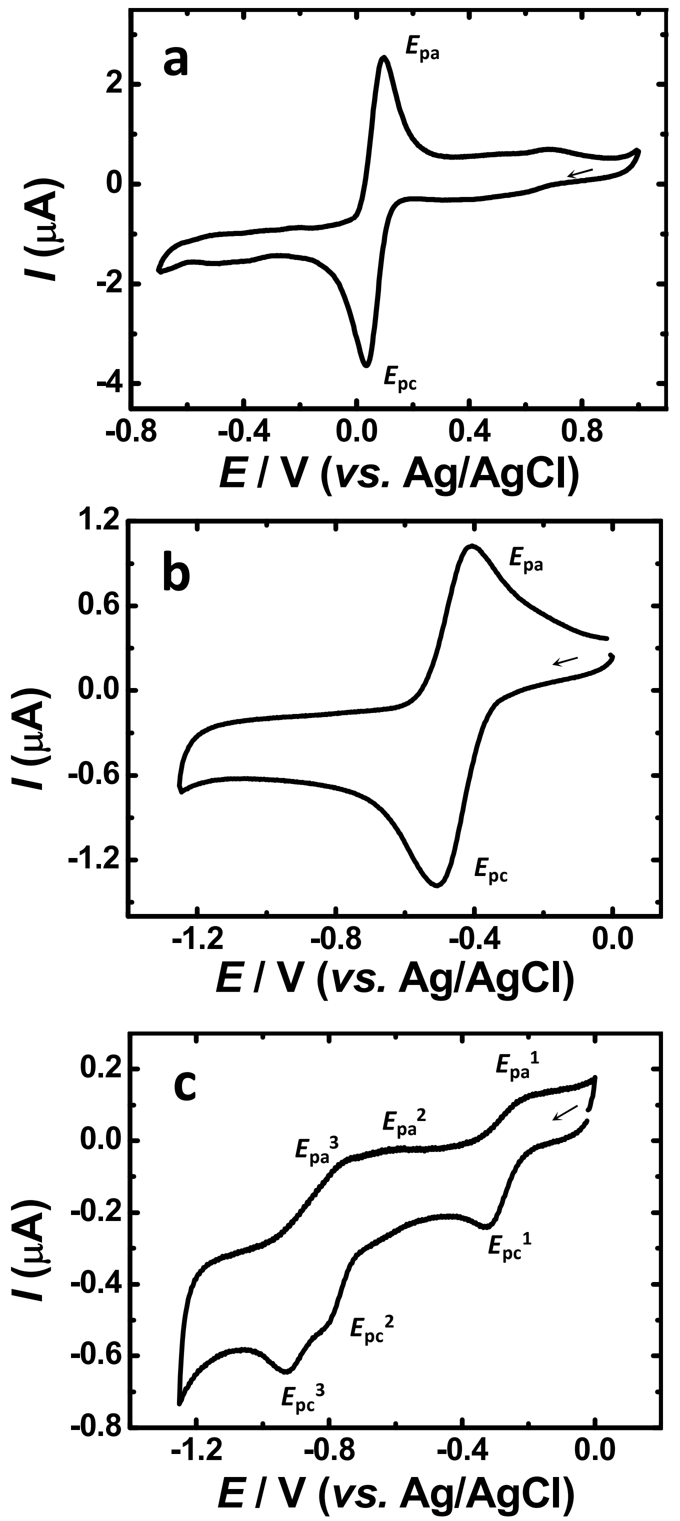

Fig. SI9 Cyclic voltammograms of 2.0 mM of TRYP-SO_3H in: (**a**) pH = 0 (1.0 M H_2SO_4) solution, (**b**) pH = 7 (1.0 M KCl) solution and (**c**) pH = 13 (1.0 M NaOH) solution, with saturated N_2, υ = 50 mV s^{-1}.

Table SI2. Solubility of $K_4[Fe(CN)_6].3H_2O$, BQDS and TRYP-SO_3H in 1.0 M KCl at T = 293 K.

Active Material	Solubility (M)
**$K_4[Fe(CN)_6].3H_2O$**	0.812 - 0.787
**BQDS**	1.280 - 1.185
**TRYP-SO_3H**	0.122 - 0.108

Table SI3. Calculation of the diffusion coefficient (*D*) for $K_4[Fe(CN)_6]$ in 1.0 M KCl solution.

Slope	*n*	*F* (C mol^{-1})	A (cm^2)	C° (mol cm^{-3})	R (J K^{-1} mol^{-1})	T (K)	*D* (cm^2 s^{-1})	Average *D* (cm^2 s^{-1})
1.38×10^{-5}	1	96485	0.02	1.00×10^{-6}	8.314	293	6.47×10^{-6}	6.63×10^{-6}
-1.41×10^{-5}							6.79×10^{-6}	

Table SI4. Calculation of the diffusion coefficient (*D*) for BQDS in 1.0 M KCl solution.

Slope	*n*	*F* (C mol^{-1})	A (cm^2)	C° (mol cm^{-3})	R (J K^{-1} mol^{-1})	T (K)	*D* (cm^2 s^{-1})	Average *D* (cm^2 s^{-1})
1.30×10^{-5}	2	96485	0.02	1.00×10^{-6}	8.314	293	7.24×10^{7}	5.38×10^{-7}
-9.08×10^{-6}							3.51×10^{-7}	

Table SI5. Calculation of the diffusion coefficient (*D*) for TRYP-SO_3H in 1.0 M KCl solution.

Slope	*n*	*F* (C mol^{-1})	A (cm^2)	C° (mol cm^{-3})	R (J K^{-1} mol^{-1})	T (K)	*D* (cm^2 s^{-1})	Average *D* (cm^2 s^{-1})
4.25×10^{-6}	2	96485	0.02	1.00×10^{-6}	8.314	293	7.68×10^{-8}	6.98×10^{-8}
-3.84×10^{-6}							6.28×10^{-8}	

Table SI6. Calculation of the electron transfer rate (k_o) for $K_4[Fe(CN)_6]$ in 1.0 M KCl solution.

υ (mV s^{-1})	△Ep (mV)	Ψ^a	k_o ($\times10^{-3}$ cm s^{-1})	Average k_o (cm s^{-1})
10	74	1.830	5.3	
20	74	1.830	7.4	
30	74	1.830	9.1	
40	74	1.830	10.5	
50	74	1.830	11.8	
60	74	1.830	12.9	1.24×10^{-2}
70	74	1.830	13.9	
80	74	1.830	14.9	
90	74	1.830	15.8	
100	74	1.830	16.6	

[a]The value was obtained from Fig. 2 according to Nicholson`s work.[1]

Table SI7. Calculation of the electron transfer rate (k_o) for BQDS in 1.0 M KCl solution.

υ (mV s^{-1})	△Ep (mV)	Ψ^a	k_o ($\times10^{-3}$ cm s^{-1})	Average k_o (cm s^{-1})
10	510	0.100	0.366	
20	543	0.100	0.346	
30	565	0.100	0.327	
40	583	0.100	0.306	
50	572	0.100	0.283	
60	599	0.100	0.259	2.71×10^{-4}
70	615	0.100	0.231	
80	605	0.100	0.200	
90	578	0.100	0.164	
100	591	0.100	0.116	

[a]The value was obtained from Fig. 2 according to Nicholson`s work.[1]

Table SI8. Calculation of the electron transfer rate (k_o) for TRYP-SO_3H in 1.0 M KCl solution.

υ (mV s^{-1})	△Ep (mV)	Ψ^a	k_o ($\times 10^{-3}$ cm s^{-1})	Average k_o (cm s^{-1})
10	117.0	0.411	0.542	
20	117.0	0.411	0.514	
30	114.0	0.334	0.394	
40	114.5	0.411	0.453	
50	114.0	0.334	0.341	
60	111.0	0.411	0.383	3.62×10^{-4}
70	114.0	0.334	0.278	
80	111.0	0.411	0.297	
90	114.0	0.334	0.197	
100	118.0	0.411	0.170	

[a]The value was obtained from Fig. 2 according to Nicholson`s work.[1]

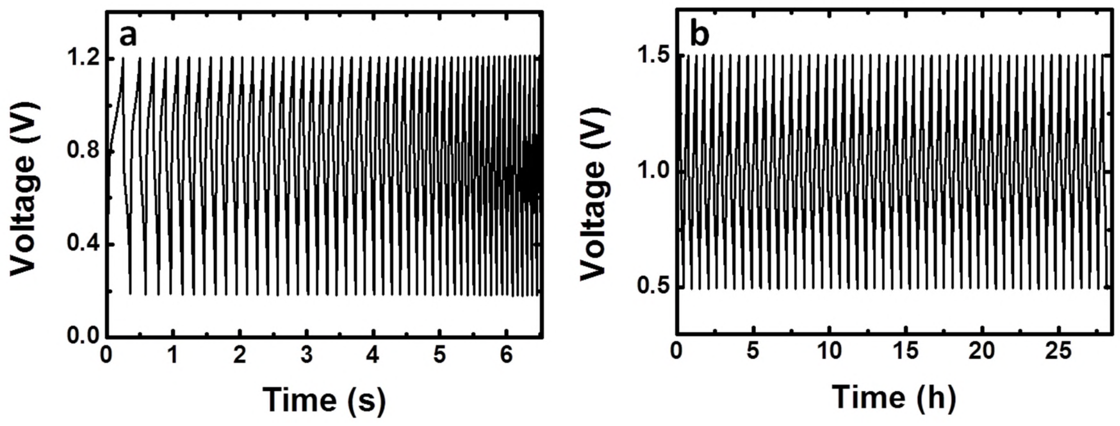

Fig. SI10 Galvanostatic charge-discharge curves of neutral pH aqueous organometallic/organic RFB single cells using 5.0 mM of TRYP-SO_3H in 1.0 M KCl solution as supporting electrolyte measured at the 50th cycle. **a** 10.0 mM of $K_4[Fe(CN)_6]\cdot 3H_2O$ as catholyte. **b** 5.0 mM of BQDS as catholyte.

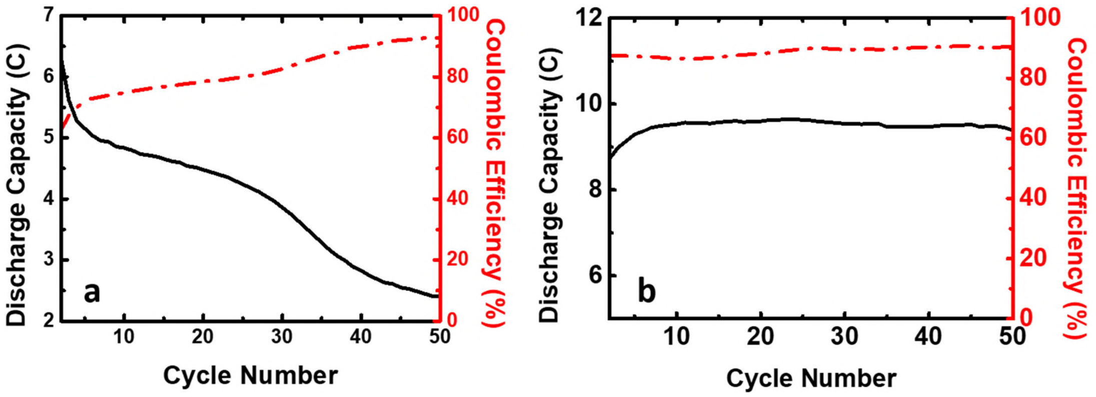

Fig. SI11 Discharge capacity (black line) and coulombic efficiency (red dash dot line) *vs* cycling numbers plots of neutral pH aqueous organometallic and all-organic active materials for RFB single cells using 5.0 mM of TRYP-SO_3H in 1.0 M KCl solution as supporting electrolyte. **a** 10.0 mM of $K_4[Fe(CN)_6]\cdot 3H_2O$ as catholyte. **b** 5.0 mM of BQDS as catholyte.

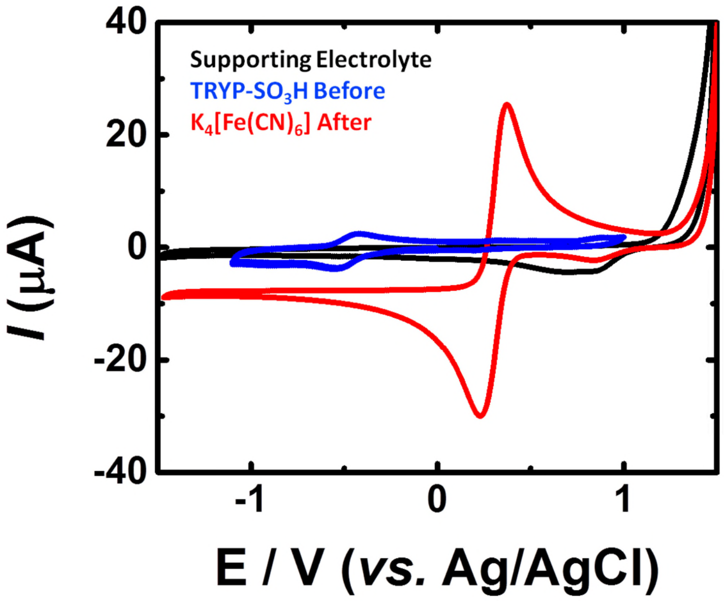

Fig. SI12 Cyclic voltammetry curves comparison between the anolyte (TRYP-SO_3H) before (blue trace) and catholyte ($K_4[Fe(CN)_6]$) after (red trace) full cell test using 1.0 M KCl as supporting electrolyte. Solutions with saturated N_2, υ = 50 mV s^{-1}.

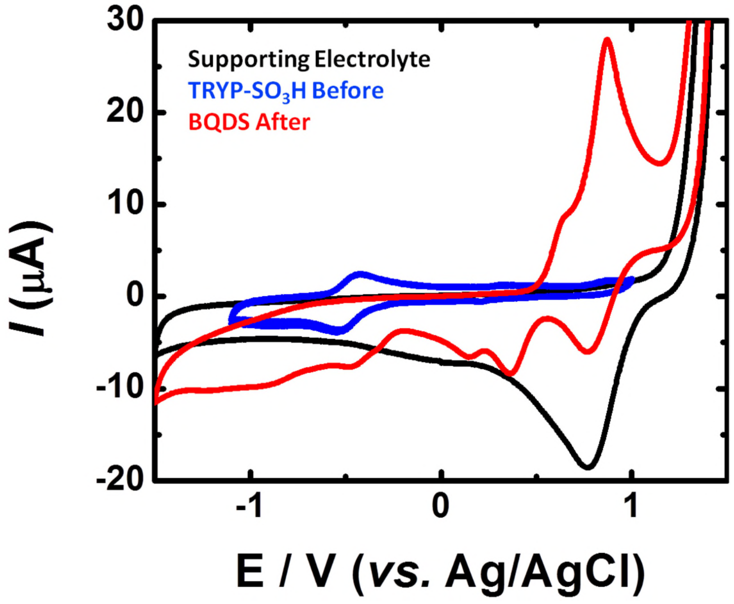

Fig. SI13 Cyclic voltammetry curves comparison between the anolyte (TRYP-SO_3H) before (blue trace) and catholyte (BQDS) after (red trace) full cell test using 1.0 M KCl as supporting electrolyte. Solutions with saturated N_2, υ=50 mV s^{-1}.

Supplementary References

1 Nicholson, R. S. Theory and Application of Cyclic Voltammetry for Measurement of Electrode Reaction Kinetics. *Anal. Chem.* **37**, 1351-1355, doi:10.1021/ac60230a016 (1965).
